# Cross-sector surveys assessing perceptions of key stakeholders towards barriers, concerns and facilitators to the appropriate use of adaptive designs in confirmatory trials

**DOI:** 10.1186/s13063-015-1119-x

**Published:** 2015-12-23

**Authors:** Munyaradzi Dimairo, Steven A. Julious, Susan Todd, Jonathan P. Nicholl, Jonathan Boote

**Affiliations:** ScHARR, Regent Court, University of Sheffield, 30 Regent Street, S1 4DA, Sheffield, UK; Department of Mathematics and Statistics, University of Reading, Whiteknights, Reading RG6 6AX UK; Centre for Research in Primary and Community Care, University of Hertfordshire, Hatfield, AL109AB Hertfordshire UK

**Keywords:** Adaptive designs, flexible designs, barriers, surveys, confirmatory trials, Phase 3, clinical trials, early stopping, interim analyses

## Abstract

**Background:**

Appropriately conducted adaptive designs (ADs) offer many potential advantages over conventional trials. They make better use of accruing data, potentially saving time, trial participants, and limited resources compared to conventional, fixed sample size designs. However, one can argue that ADs are not implemented as often as they should be, particularly in publicly funded confirmatory trials. This study explored barriers, concerns, and potential facilitators to the appropriate use of ADs in confirmatory trials among key stakeholders.

**Methods:**

We conducted three cross-sectional, online parallel surveys between November 2014 and January 2015. The surveys were based upon findings drawn from in-depth interviews of key research stakeholders, predominantly in the UK, and targeted Clinical Trials Units (CTUs), public funders, and private sector organisations. Response rates were as follows: 30(55 %) UK CTUs, 17(68 %) private sector, and 86(41 %) public funders. A Rating Scale Model was used to rank barriers and concerns in order of perceived importance for prioritisation.

**Results:**

Top-ranked barriers included the lack of bridge funding accessible to UK CTUs to support the design of ADs, limited practical implementation knowledge, preference for traditional mainstream designs, difficulties in marketing ADs to key stakeholders, time constraints to support ADs relative to competing priorities, lack of applied training, and insufficient access to case studies of undertaken ADs to facilitate practical learning and successful implementation. Associated practical complexities and inadequate data management infrastructure to support ADs were reported as more pronounced in the private sector. For funders of public research, the inadequate description of the rationale, scope, and decision-making criteria to guide the planned AD in grant proposals by researchers were all viewed as major obstacles.

**Conclusions:**

There are still persistent and important perceptions of individual and organisational obstacles hampering the use of ADs in confirmatory trials research. Stakeholder perceptions about barriers are largely consistent across sectors, with a few exceptions that reflect differences in organisations’ funding structures, experiences and characterisation of study interventions. Most barriers appear connected to a lack of practical implementation knowledge and applied training, and limited access to case studies to facilitate practical learning.

**Electronic supplementary material:**

The online version of this article (doi:10.1186/s13063-015-1119-x) contains supplementary material, which is available to authorized users.

## Background

*“… there can be no objection to the use of data, however meagre, as a guide to action required before more can be collected ….” Thompson, 1933* [1]

The role of clinical trial adaptation has started to come to the fore in recent years, with numerous methodological developments over the past 40 years aimed at improving efficiency in the design and conduct of clinical trials [2, 3]. Researchers and policymakers are now considering adaptive designs (ADs) more often than before, and the use of ADs is increasing [4–6]. However, one could still argue that ADs, particularly in confirmatory trials, remain underutilised.

Recent research has investigated barriers and opportunities to the use of ADs, with emphasis on the pharmaceutical trial perspective [4, 6–8], the US setting [9, 10], and publicly funded early phase trials in the UK [11]. Some of these previous studies have been informal in nature, and some important barriers to successful implementation could potentially have been overlooked. Furthermore, the attitudes of public funders towards the use of ADs have not been formally explored, and little focus has been directed to confirmatory trials in the UK setting. To address these gaps in previous research, we undertook multi-disciplinary and cross-sector in-depth interviews of key stakeholders in clinical trials research to inform the design of subsequent surveys [12]. Interviews were undertaken with a range of researchers including public funding panel chairs and members, statisticians, leaders of UK Clinical Trials Units (CTUs), chief investigators, regulators, health economists, and data monitoring committee members.

Based upon themes generated from the interview findings [12], we conducted quantitative surveys aimed at public and private sector researchers, and public funders, predominantly in the UK. The main objectives of the surveys were as follows:

1. Investigate perceived barriers to and concerns regarding the use of ADs in confirmatory trials and gauge the opinions of key stakeholders, such as public funders;

2. Rank the barriers and concerns in order of perceived importance for prioritisation and compare and contrast these rankings between public and private sector researchers;

3. Investigate the types of ADs being implemented in practice in confirmatory trials; and

4. With a focus on publicly funded trials, explore ways to address perceived barriers and concerns and propose recommendations in cases where ADs may potentially offer added benefits to standard trial designs.

## Methods

Between November 2014 and January 2015, we conducted three cross-sectional, parallel, quantitative online surveys tailored for UK CTUs, private sector organisations, and public funders, exploring perceptions towards and potential facilitators to the appropriate use of ADs in confirmatory trials.

### Surveys’ sampling frames

This research paid attention to researchers both in the private and public sector as a collaborative platform for addressing cross-sector roadblocks to the appropriate use of ADs. In addition, the conduct of clinical trials research and approval of effective and safe healthcare interventions into medical practice is a complex process involving various key stakeholders, such as funders/sponsors, researchers and policymakers. Hence, prospective participants were identified as follows:

The United Kingdom Clinical Research Collaboration (UK CRC) comprises a network of registered CTUs with expertise to coordinate and support high quality conduct of clinical trials [13]. Consequently, major UK public funders such as the National Institute for Health Research (NIHR) and the Medical Research Council (MRC) require, as part of their funding policy, the involvement of UK CRC-registered CTUs with the vision to improve quality in the conduct and delivery of publicly funded clinical trials. Overall, there were 55 registered CTUs (2013/2014) across the UK [13], which we contacted to take part in our research.

The NIHR is one of the major UK public funders of medical research, contributing about a third of total research funding – spending over £1 billion in 2013/2014 [14]. Within the NIHR programme, the Health Technology Assessment (HTA) is the largest funding stream for the commissioning of independent research, which includes predominantly confirmatory trials to assess clinical and cost effectiveness, and the broader impact of healthcare interventions that have been tailored for the National Health Service (NHS) [15]. The HTA programme has four boards supported by five advisory panels and a priority group, some of whose members are publicly contactable [16]. There are other NIHR funding streams supporting a smaller proportion of confirmatory trials, such as the Efficacy Mechanism and Evaluation (EME), and Research for Patient Benefit (RfPB). In acknowledging the crucial role of charity funders in clinical trials research and to capture diverse views, Cancer Research UK (CR UK) advisory panel members were also contacted because it is one of the largest UK charities funding confirmatory trials. In addition, huge opportunities to use adaptive designs are perceived in therapeutic areas such as oncology [12]. We also approached a second large UK charity funder, but unfortunately, the coordinating team was uncomfortable about us contacting their boards and panel members. Overall, 212 contactable public funding board members and advisory panel members from HTA (n = 110), EME (n = 20), RfPB (n = 40), and CR UK (n = 42) were approached to take part.

Private sector trials research is mostly conducted by pharmaceutical and biotech companies, and Contract Research Organisations (CROs). Twenty-five companies with which we had direct contact were approached to take part: pharmaceutical or biotech organisations (n = 13) and CROs (n = 12).

### Design of survey instruments

**Fig. 1:**
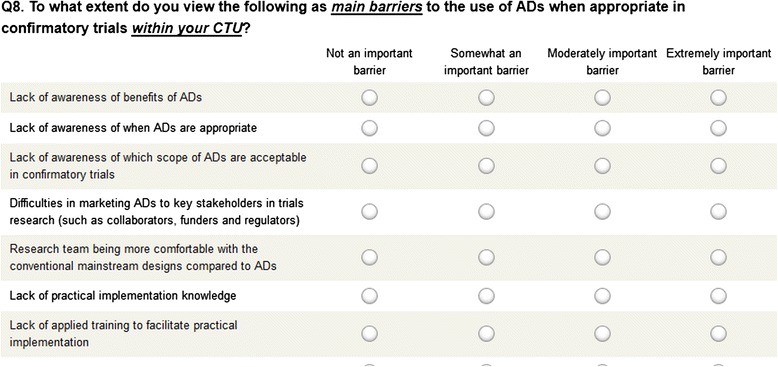
A snapshot of the UK CTUs survey instrument

A list of themes on perceived barriers, concerns, and potential facilitators was compiled based on prior findings from our in-depth interview study [12]. These themes were then grouped to develop survey instruments depending on to whom they pertained: public funders, UK CTUs, and private sector. Most of the questions were of a closed form; however, we included open-ended questions for respondents to add detailed responses where applicable. We accessed the perceptions of respondents on the importance of barriers and concerns towards, and usefulness of, potential facilitators to the appropriate use of ADs in confirmatory trials using widely accepted Likert Scales [17]. Most questions for the UK CTU and private sector surveys were phrased consistently, except on those occasions when specific questions were unique to a certain sector. Instruments were internally reviewed and piloted prior to the launch of the online surveys. Figure 1 displays a snapshot of CTU-specific questions on barriers.

### Approaching targeted participants

The Directors or designated Senior Statisticians were contacted to complete the online survey instrument tailored for UK CTUs. Only one response per CTU representative was permitted. Two rounds of email invitations with an information sheet were sent through the UK CRC-registered CTU network of Directors and Senior Statistician representatives. A third round of personalised emails were directly sent to some non-responders (n = 21) of the previous two rounds.

Public funding boards and panels chairs and ordinary and lay members were approached through email to complete an online survey tailored for public funders. One round of invitation emails was sent to panel chairs and vice chairs, and some panel members through the NIHR programme coordinators. Some members were directly contacted by personalised emails using online, publicly accessible contact details.

For the private sector; two rounds of emails were directly sent to trial Research Leaders or designated Principal or Senior Statisticians from 25 organisations with whom we had contact. Multiple responses from the organisations with several trial research groups were permitted where applicable.

All cross-sector participants were given between 3 and 8 weeks to complete the online surveys.

### Statistical analysis and reporting

Descriptive statistics were generated, aided by forest plots and clustered bar charts to display respondent perceptions. A Rating Scale Model for ordered response items [18] was employed to rank the perceived importance of barriers and concerns as characterised by the ‘difficulty’ parameter using the RUMM2030 software [19]. A respondent’s log odds of choosing a higher category of an item on an importance (or concern) scale over the previous adjacent category was modelled as a function of the ability of the respondents, perceived level of importance (or concern) attached to an item, and threshold parameters of items as follows [18]:

$$ \ln \left(\frac{p_{nik}}{p_{ni\left(k-1\right)}}\right)={\theta}_n-\left({\delta}_i+{\tau}_k\right), $$

where *p*_*nik*_ is the probability of a respondent *n* with ability *θ*_*n*_ choosing category *k* for an item *i*; *k* = 0,1,2,…; *m* represents the ordered choices, and *m* is the number of item steps; *δ*_*i*_ is the ‘difficulty’ of item *i*, which is the importance location parameter of interest; *τ*_*k*_ is the threshold parameter corresponding to choice *k* in item *i*; and *θ*_*n*_ and *τ*_*k*_ are nuisance parameters.

The research utilised a recent review of existing guidance on the reporting of survey research and attempted to use relevant items to enhance its reporting [20]. To facilitate interpretation and consistency, barriers and concerns to the use of ADs are presented in order of perceived importance. Proportions of item responses, estimates of the importance parameters with associated 95 % confidence intervals (CIs), and item rank are presented.

### Ethical approval

This study received favourable ethical approval (0676) from the School of Health and Related Research (ScHARR) Ethics Committee at the University of Sheffield. Participants were only permitted to complete the survey after consenting, which was the opening question on all survey instruments.

## Results

### Response rates

We observed a UK CTU crude response rate of 55 % (30/55). In addition, 46 % (25/55) of respondents completed all key questions (barriers, concerns, and potential facilitators). Approximately 68 % (17/25) of the organisations responded to the private sector survey. Of these, 52 % (13/25) responded to all key survey questions. Approximately 41 % (86/212) of the respondents completed the online survey tailored for public funders. However, the response rate to all key questions was around 30 % (64/212). Non-response feedback from two UK CTUs and six public funders cited a lack of basic understanding of ADs to meaningfully contribute to the survey as the main reason for non-participation.

### Baseline characteristics of respondents

**Fig. 2:**
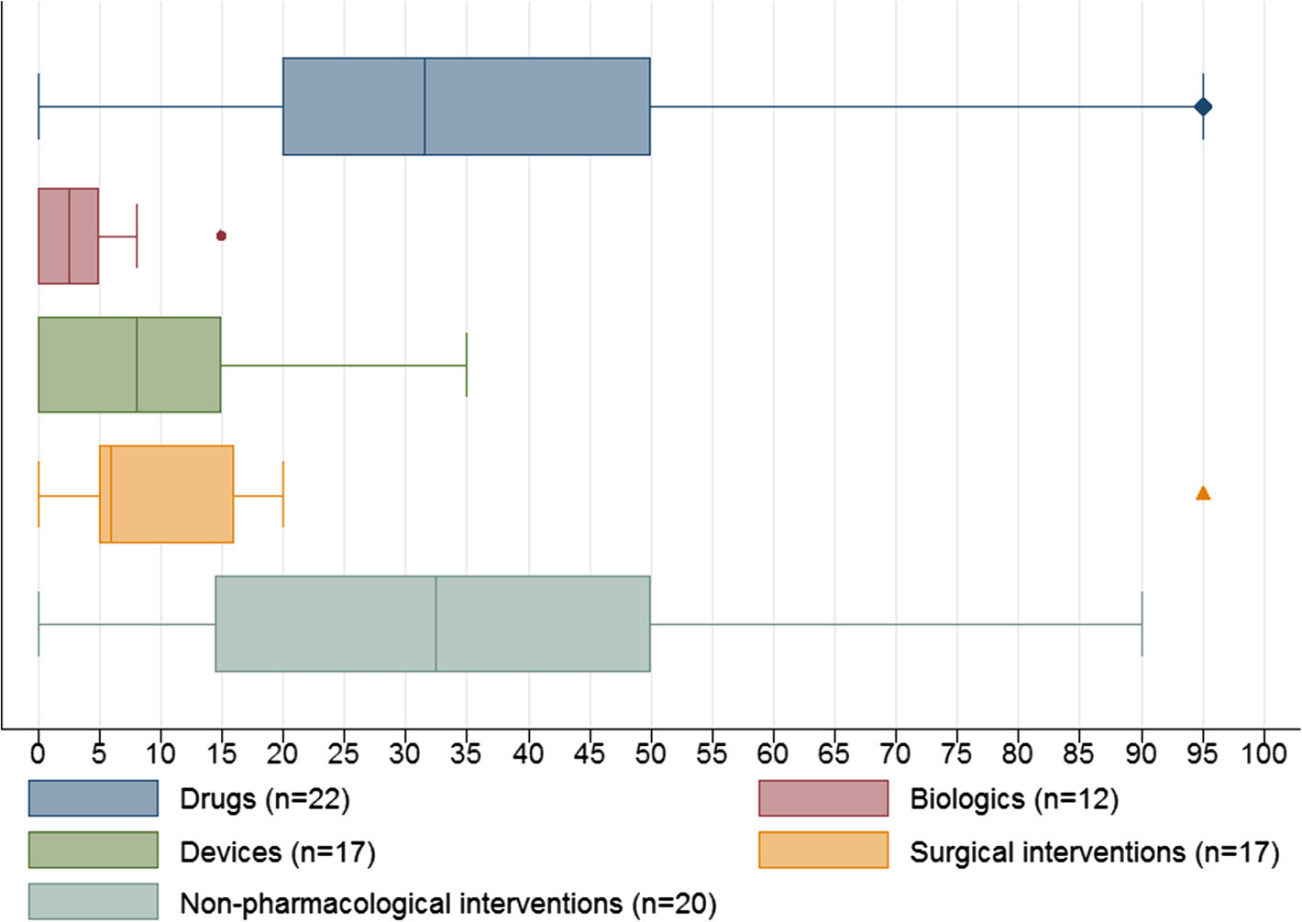
The distribution of the nature of interventions investigated by UK Clinical Trials Unit (CTU) respondents

Of the 30 UK CTUs respondents, 10(33 %) and 18(60 %) were Directors and Senior Statisticians respectively, and two did not state their role. CTUs covered a wide geographical area across the UK and represented many therapeutic areas of clinical trials research. Overlapping and diverse therapeutic areas of research include oncology (n = 13), mental health (n = 11), primary care (n = 9), public health (n = 9), musculoskeletal (n = 8), respiratory (n = 8), cardiovascular (n = 7), diabetes (n = 7), health services (n = 7), emergency medicine (n = 6), infectious (n = 2), rare or orphan diseases (n = 2), perinatal medicine (n = 1), surgical interventions (n = 1), and other (n = 3). Figure 2 displays the approximate distribution of the study interventions as a percentage of the total number of trials based on complete data reported. In addition, the distribution of the trials requiring regulatory approval (such as from the MHRA, EMA, or FDA) beyond standard ethics had a median (IQR) of 50 % (16 % to 80 %), based on 23 complete responses.

The expertise of public funder respondents in clinical trials research was diverse and overlapping, representing the composition of public funding boards and advisory panels. These included trial statisticians 11(13 %), chief investigators 40(47 %), trial methodologists 20(23 %), trial management experts 6(7 %), clinical experts 23(27 %), health economists 9(10 %), Independent Data Monitoring Committee 33(38 %) and Trial Steering Committee members 24(28 %), CTU directors 12(14 %), patient representatives 7(8 %), and other 6(7 %). Ordinary members and Chairs or Vice Chairs of boards or panels constituted 63(73 %) and 10(12 %), respectively. Most members (65 %) had served for less than 5 years on their current funding boards or panels. Of the 17 industry respondents, 9(56 %) and 7(44 %) were from pharmaceutical or biotech organisations and CROs, respectively, and one did not state the nature of their organisation. Private sector respondents were predominantly Lead Statisticians in the UK 13(81 %).

### UK CTU perceptions of barriers to ADs use in confirmatory trials

**Fig. 3:**
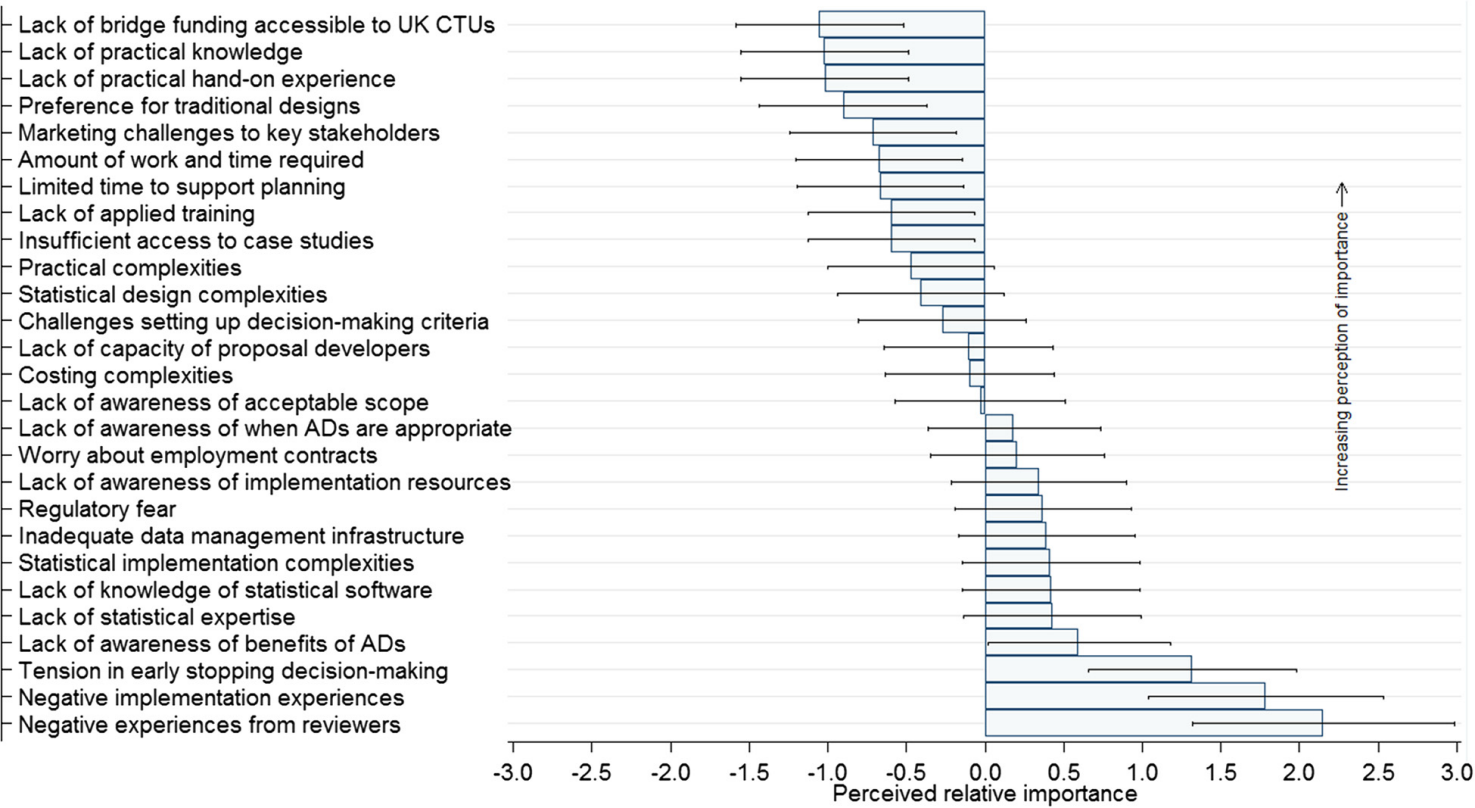
Ranked perceptions of UK Clinical Trials Units (CTUs) on the importance of barriers to adaptive designs (ADs) use in confirmatory trials

The distribution of respondents’ opinions and rank of barriers, based on estimates of the perceived relative importance parameters with associated 95 % CIs, from the Rating Scale Model are displayed in Fig. 3. The more negative or smaller the relative importance parameter, the more important respondents perceived the barrier. Supplementary summary data with detailed description of survey questions relating to the barrier items presented in Fig. 3 are provided (see Additional file 1).

The lack of bridge funding accessible to CTUs to support the design work of complex and time-consuming ADs was perceived as the top-ranked obstacle impeding the routine use of Ads and was reported by 8(32 %) and 12(48 %) respondents as an ‘extremely’ or and ‘at least moderately’ important barrier, respectively. The lack of practical implementation knowledge and hands-on experience was the second highest ranked barrier, with 6(24 %) ‘extremely’ and 15(60 %) ‘at least moderately’ important rankings. The opinions of respondents suggested that research teams within the UK CTUs have a strong preference for traditional mainstream designs which they know well and feel uncomfortable supporting ADs even when they are appropriate. Only 3(12 %) respondents did not view it as an important barrier, whereas 6(24 %) and 12(48 %) respondents reported it as an ‘extremely’ and ‘at least moderately’ important barrier, respectively (see Additional file 1).

Thirteen (52 %) respondents reported difficulties faced by trialists in marketing ADs to key stakeholders in trials research, such as clinical collaborators, funders, and regulators as ‘at least a moderately’ important barrier. The amount of time and effort required to support the design of ADs, and the time constraints relative to competing priorities of the traditional mainstream designs was reported as ‘at least an important’ barrier by 12(48 %) respondents. The lack of applied training and insufficient access to case studies on ADs to facilitate practical learning and implementation were reported among the top-ranked barriers. Statistical complexities such as simulations work as part of design, practical implementation complexities and difficulties faced by clinical trialists in setting up acceptable planned decision-making criteria to guide the adaptation were among some of the middle-ranked barriers.

Barriers reported as ‘not at all’ important by many respondents were negative experiences based on funders’ or reviewers’ comments (76 %), negative implementation experiences (76 %), early stopping decision-making tensions among key decision-makers (60 %), and lack of awareness of benefits of ADs (48 %).

### UK public funder perceptions on barriers to the use of ADs in confirmatory trials

**Fig. 4:**
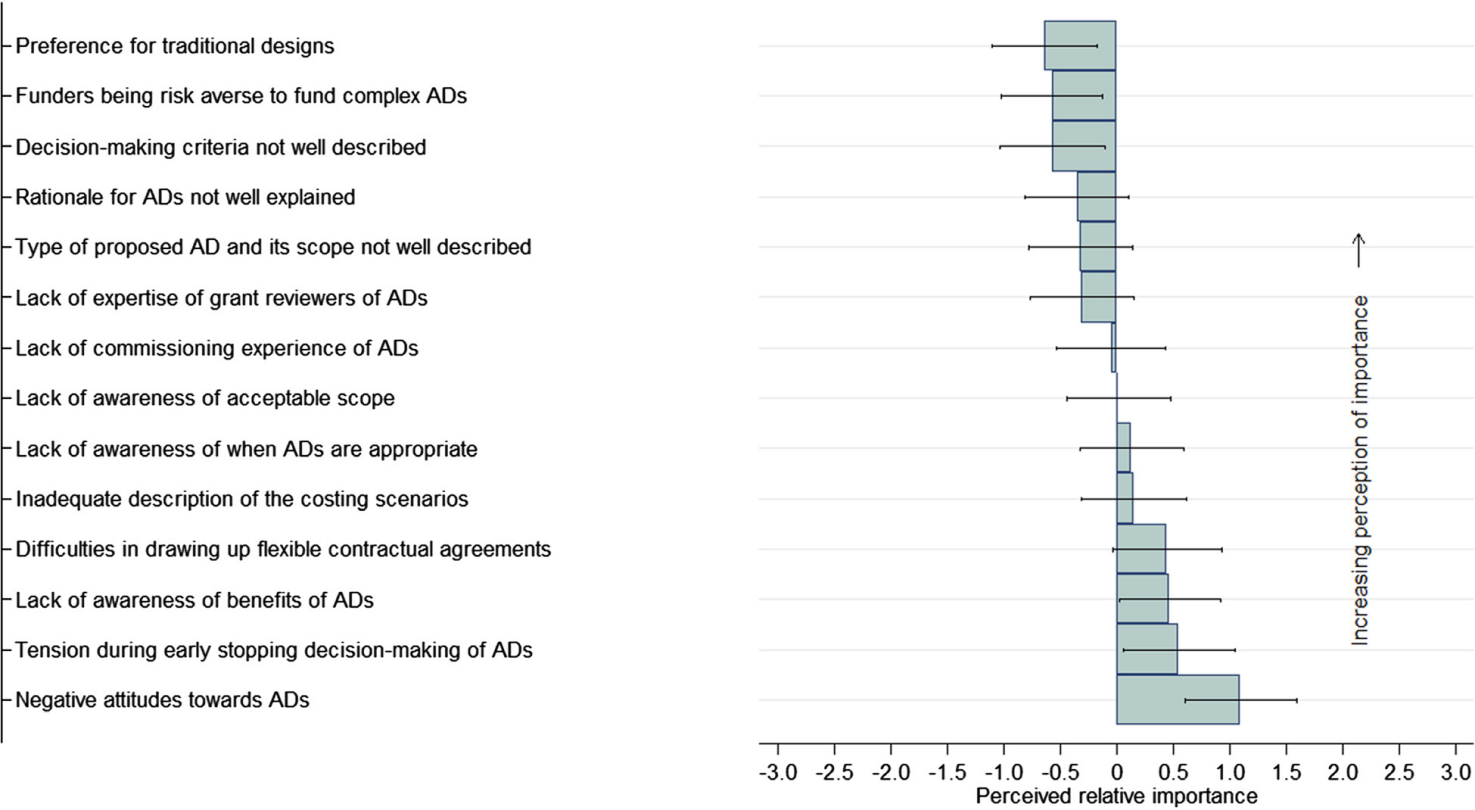
Ranked perceptions of UK public funders on the importance of barriers to adaptive designs (ADs) use in confirmatory trials

Public funder respondents ranked the importance of most barriers considered with a small degree of differentiation (Fig. 4). Supplementary summary data with detailed description of survey questions relating to barrier items presented in Fig. 4 are provided (see Additional file 2). The preference of public funders for traditional mainstream designs over ADs and their risk-averse attitude to fund projects associated with a high degree of financial uncertainty were among the most important barriers reported. Researchers’ inadequate description of the rationale for using an AD and decision criteria to guide the AD in grant proposals were reported as ‘at least moderately’ important barriers by 60 % and 53 % of respondents, respectively, thus making the review process challenging. The dearth of expert proposal reviewers to provide advisory support to funders during their grant commissioning process was selected by 32(55 %) and 11(19 %) of respondents, as a ‘moderately’ or ‘extremely’ important barrier, respectively. Inadequate descriptions by the researchers of the proposed ADs and the scope of these descriptions in the grant applications were also ranked among the top-ranked barriers. Some of the middle-ranked barriers were lack of commissioning experience of AD-related research, and lack of awareness of acceptable scope of ADs and when they are appropriate in the confirmatory setting.

Challenges faced by funders in drawing up contractual agreements that are suitable to support ADs were reported by 38 % and 10 % of respondents to be a ‘moderately’ or ‘extremely’ important barrier. The lowest ranked of the perceived barriers were early trial stopping decision-making tensions among key decision-makers and negative attitudes towards ADs by some of the funding boards and panel members.

### Private sector perceptions of barriers to the use of ADs in confirmatory trials

**Fig. 5:**
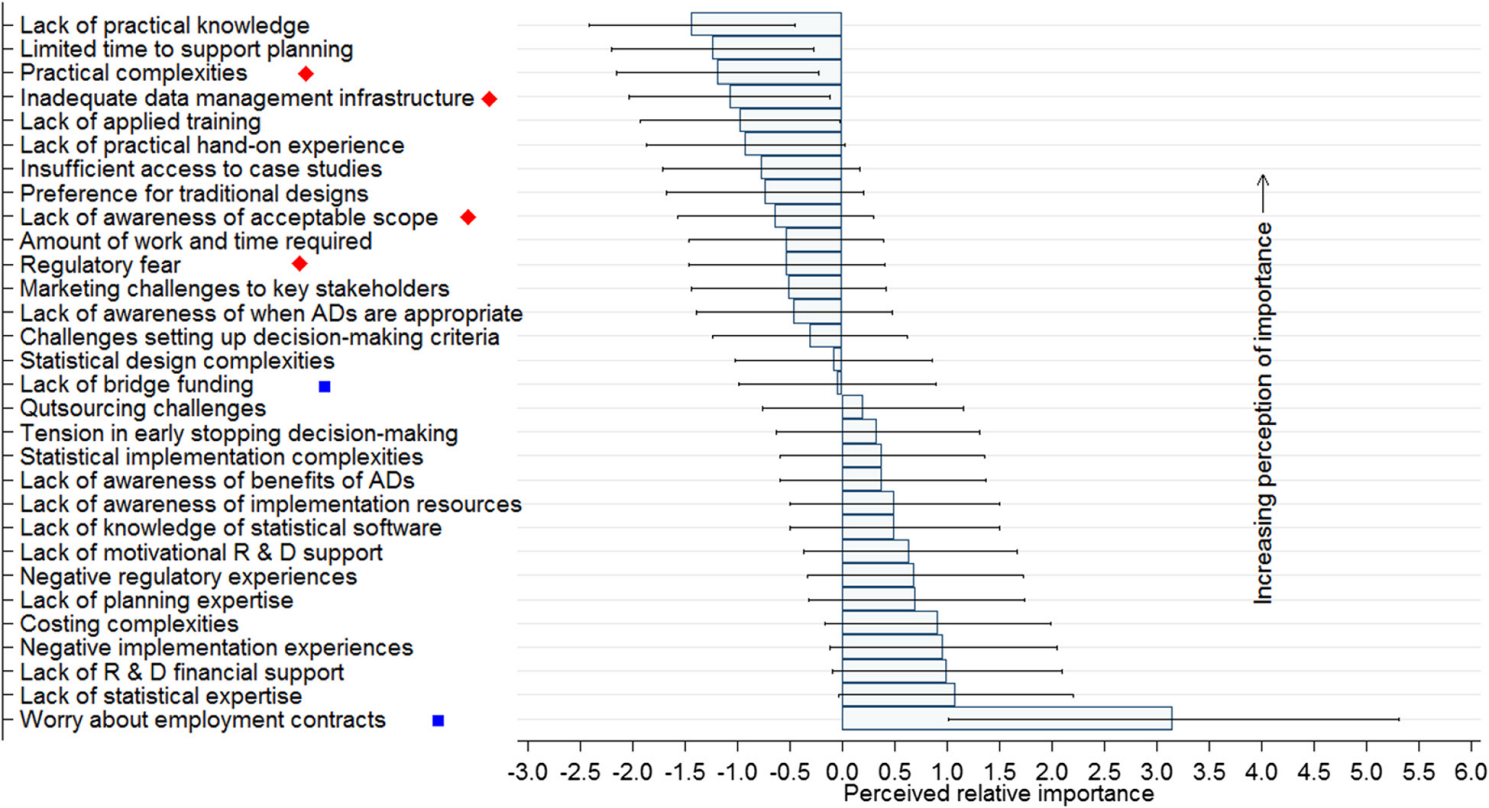
Ranked perceptions of the private sector organisations on important barriers to adaptive designs (ADs) use in confirmatory trials. Red diamonds indicate barriers that were ranked higher by the private sector than UK Clinical Trials Units (CTUs). Blue squares indicate barriers that were ranked higher by the UK CTUs than the private sector

In general, the perceptions of UK CTUs and the private sector on the importance of different barriers are consistent; however, there are a few exceptions (Figs. 3 and 5). For instance, complexities during practical implementation, inadequate data management infrastructure, and fear of risking regulatory approval appear to be very prominent in the private sector (marked in red diamonds on Fig. 5). In contrast, the lack of bridge funding to support developmental design work and worry about research staff employment contracts when trials are stopped early were highly and middle rated in the public sector, respectively (marked in blue squares on Fig. 5). The top-ranked barriers reported to be ‘at least moderately’ important were as follows (see Additional file 3): the dearth of practical implementation knowledge 9(69 %); time constraints to support the planning of complex ADs relative to competing priorities of traditional mainstream designs 6(46 %); associated practical complexities during implementation of ADs 9(69 %); inadequate data management infrastructure for timely data capturing, cleaning, and processing to support ADs 5(42 %); the dearth of applied training to facilitate practical implementation 9(69 %) and lack of hands-on practical experience 8(62 %); limited access to case studies of the few ADs that have been implemented to facilitate practical learning 6(46 %); and research teams being more comfortable with traditional mainstream designs 8(62 %).

The middle ranked important barriers reported with little degree of separation between them were the lack of awareness of acceptable scope of ADs in confirmatory trials; enormous amount of time and effort required during planning; fear of regulatory reluctance and risk of jeopardising chances of approval; difficulties faced in marketing ADs to key stakeholders; the lack of awareness of when ADs are appropriate; and difficulties faced in setting up upfront, acceptable decision-making criteria to guide trial adaptation (Fig. 5, Additional file 3).

Barriers reported as unimportant by a sizable number of respondents were a lack of awareness of implementation resources 6(46 %), lack of knowledge of existing AD-related statistical software 6(46 %), lack of motivational support from Research and Development (R & D) 6(46 %), negative regulatory experiences 5(38 %), lack of expertise to support planning 5(38 %), costing complexities during planning 6(46 %), negative experiences during implementation 7(54 %), insufficient R & D financial support to invest in AD infrastructure 8(62 %), the dearth of statistical expertise to support ADs 8(62 %), and worry about staff employment contracts when trials are stopped early 10(77 %).

### Cross-sector perceived concerns about the use of ADs in confirmatory trials

The early stopping of trials (or treatment arms) for futility - as soon as there is sufficient evidence of no benefit - was the least ranked concern across sectors (see Additional file 4). Concerns about the robustness of ADs in decision-making and the acceptability of findings to change medical practice when trials are stopped early appeared more pronounced among public funder respondents. The early stopping of trials for efficacy or non-inferiority were top-ranked concerns reported by UK CTUs and private sector respondents, respectively. The fear of introducing operational bias, impact of ADs on other important study objectives (such as safety and health economic evaluation) when trials are stopped early, and the potential of population drift as a result of the AD and its implications on the interpretation of findings were viewed with varying degrees of concern among cross-sector respondents. Supplementary summary data on cross-sector concerns about the use of ADs in confirmatory trials and detailed description of survey questions are provided (see Additional file 4).

### Organisational priority to the use of ADs in confirmatory trials

When respondents were asked to rate the level of organisational priority they give to the use of ADs and/or research on ADs–related methods within the next 5 to 10 years, 15(50 %) of the UK CTUs selected it as a ‘medium priority’ and just 3(10 %) as a ‘high priority’. In contrast; 5(29 %), 4(24 %) and 4(24 %) private organisations selected it as a ‘medium priority’, ‘high priority’, and an ‘essential priority’, respectively.

Only 2(7 %) UK CTUs and 3(18 %) of private sector organisations reported having an AD-related Working Group within their organisation. The willingness to ‘definitely consider’ the use of ADs in future confirmatory trials, when appropriate, was expressed among 16(53 %) UK CTUs and 11(65 %) private sector organisations.

Forty-five (55 %) of the public funder respondents rated their boards or panels priorities on funding themes on confirmatory ADs in the next 5 to 10 years as at least a ‘medium priority’, 19(22 %) as ‘high priority’ and only 4(5 %) as an ‘essential priority’. As a funding board or panel, only 26(30 %) reported that they had previously recommended funding a confirmatory AD grant proposal; however, 26(30 %) did not respond to the question. When asked whether they would consider recommending a confirmatory AD grant proposal for funding in the future when appropriate to address research question(s); 42(49 %) indicated that they ‘would definitely consider’, 21(24 %) ‘might or might not consider’, 1(1 %) ‘would not consider’, and 22(26 %) did not respond to the question.

### Potential facilitators to the appropriate use of ADs in confirmatory trials

**Fig. 6:**
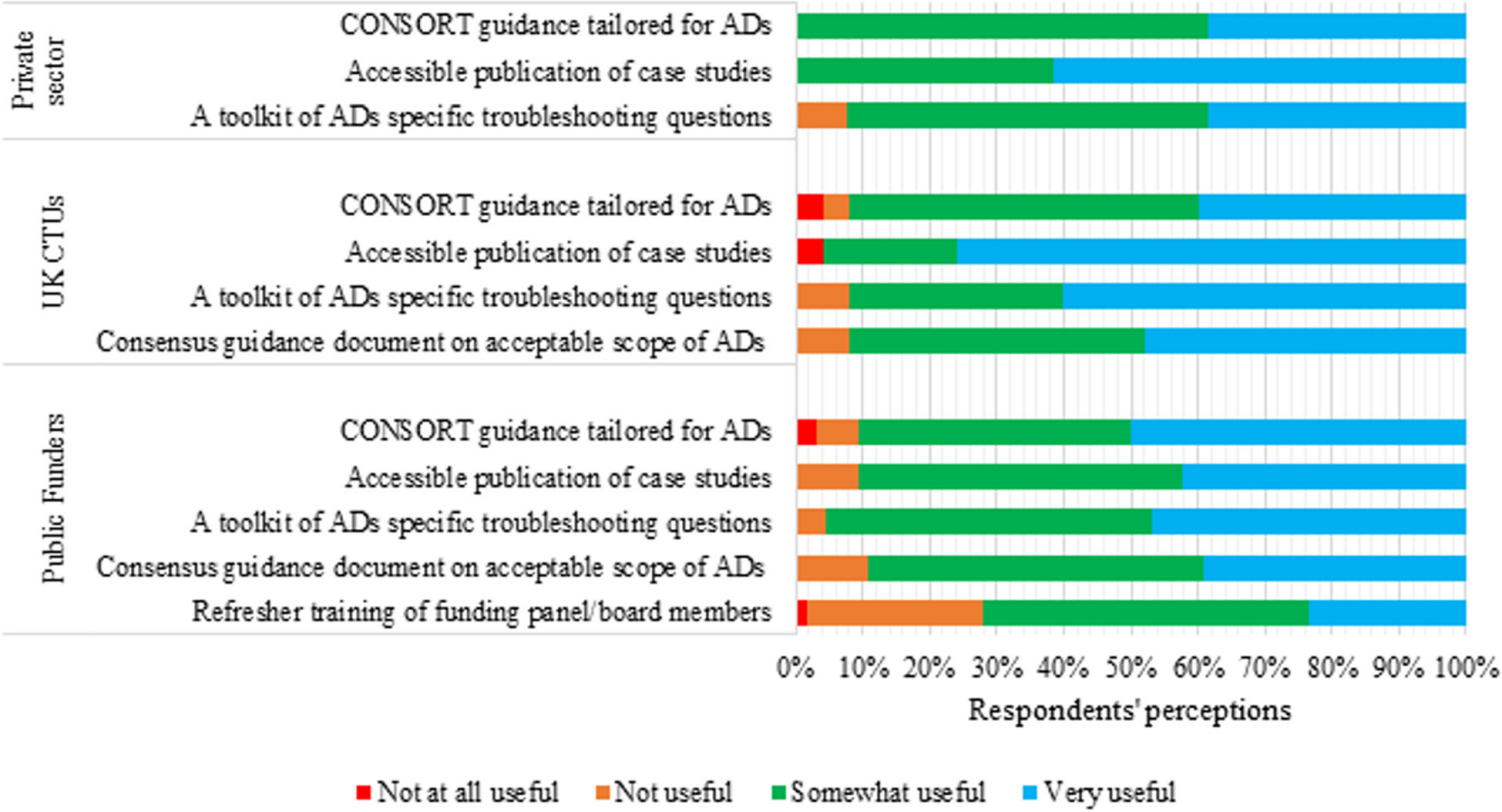
Cross-sector perceptions of potential facilitators to the use of adaptive designs (ADs) in confirmatory trials. Denominators: public funders (n = 64), UK Clinical Trials Units (CTUs) (n = 25), and private sector organisations (n = 13)

We found consistency in respondents’ perceptions across sectors regarding the usefulness of potential facilitators to enhance the appropriate use of ADs. Figure 6 summarises the respondents’ opinions. The majority of respondents reported that the availability of a troubleshooting toolkit of specific questions which clinical trialists need to ask themselves when considering the different types of ADs would be ‘somewhat useful’: 23(92 %) UK CTUs, 61(95 %) public funders, and 12(92 %) private sector organisations. The overwhelming majority of respondents reported that the access to published case studies of implemented ADs focusing on aspects such as design and rationale, implementation, regulatory and statistical challenges; lessons learned; what went wrong; and facilitators to challenges would be ‘very useful’ to researchers. Twenty-three (92 %) CTUs and 57(89 %) of the public funder respondents reported that the need for a consensus guidance document on the acceptable scope of ADs, which addressed issues tailored for publicly funded confirmatory trials, would be at least ‘somewhat useful’.

The existence of a CONSORT statement tailored for ADs was selected as at least ‘somewhat useful’ to enhance transparency and completeness in the conduct and reporting of ADs by 56(88 %) public funders, 23(92 %) CTUs, and 13(100 %) private sector organisations. A total of 46(72 %) of public funder respondents viewed that refresher training of funding boards and panel members to be familiar with AD-related issues could help them in the reviewing and commissioning process.

### Types of ADs implemented in confirmatory trials

The submission of historical AD-related grant proposals for funding considerations was reported among 13(43 %) of the UK CTUs. Historical use of at least some type of AD in confirmatory trials was reported in 27 % (8/30) of UK CTUs and 47 % (8/17) of the private sector organisations that responded to the surveys. Table 1 describes the type and scope of ADs implemented in the public and private sector. The use of ADs appears not to be widespread across sector organisations; however, there are a small number of pacesetter organisations frequently using certain types of ADs as part of their research development programs. The most commonly used AD methods across the sector include sample size re-estimation (SSR), standard two-arm group sequential design (GSD), futility analyses using stochastic curtailment methods, and operational seamless 2/3 design. Moreover, ADs with some form of futility stopping, such as dropping futile treatment arms or stopping trials, appeared popular, consistent with cross sector/disciplinary receptiveness.

**Table 1:** Distribution of the type of adaptive designs (ADs) implemented in confirmatory trials and their frequency stratified by sector

Type of AD and its description	UK Clinical Trials Units (CTUs)			Private sector		
	Number of CTUs	Number of trials	Missing responses	Number of organisations	Number of trials	Missing responses
Sample size re-estimation (SSR)	7(23 %)			7(41 %)		
Blinded SSR allowing for increase only	4	4	1	2	11	-
Blinded SSR allowing for increase or decrease	2	1	1	2	3	-
Unblinded SSR allowing for increase only	2	5	1	2	10	-
Unblinded SSR allowing for increase or decrease	2	5	1	-	-	-
Unblinded SSR based on promising zone concept	2	-	2	3	10	-
Standard two-arm Group Sequential Design (GSD)	7(23 %)			8(47 %)		
Stopping early for futility only	2	7	1	3	26	-
Stopping early for efficacy only	1	.	1	-	-	-
Stopping early for efficacy or futility	4	6	-	3	8	1
Stopping early for safety only	4	2	2	2	5	1
Stopping early for safety or futility	2	2	-	1	5	-
Stopping early for non-inferiority only	-	-	-	1	-	1
Futility analysis (outside GSD framework)	8(27 %)			5(29 %)		
Based on conditional power	5	7	1	3	3	2
Based on predictive power	2	1	1	1	-	1
Based on confidence interval of the interim effect	3	3	1	-	-	-
Operational seamless 2/3 design	7(23 %)			6(35 %)		
Dropping futile treatment arms in phase 2 only	5	5	1	3	3	1
Selecting only one promising treatment in phase 2 only	1	-	1	3	2	1
Selecting multiple promising treatments in phase 2 only	-	-	-	2	3	1
Other	2	-	2	-	-	-
Inferential seamless 2/3 design	2(7 %)			3(18 %)		
Dropping futile treatment arms in phase 2 only	2	1	1	1	2	-
Addition or dropping futile treatment arms in phase 2 only	1	.	1	-	-	-
Strictly phase 3 multi-arm multi-stage design	2(7 %)			2(17 %)		
Stopping trial for efficacy or futility or dropping futile treatment arms	1	1	-	-	-	-
Information-based GSD	-	-	-	4(24 %)	3	2
Standard GSD with SSR	-	-	-	1(6 %)	-	1
Patient enrichment or subgroup selection	2 (7 %)	.	2	2(12 %)	-	2
Response adaptive randomisation	2(7 %)	2	-	2(12 %)	2	1

Note: Denominator is based on responders; UK CTUs (n = 30) and private sector organisations (n = 17)

## Discussion

We need to emphasise that ADs are not appropriate for every trial. When contemplating the use of ADs, logistical as well as statistical considerations should be made on a trial-to-trial basis. These considerations include the accrual of the primary endpoint data in relation to the expected recruitment rate, the rationale for choosing the design, feasibility or practicalities of implementing the design in practice, and potential benefits against additional complexities in implementation. Some of the considerations are highlighted in our preceding research [12].

### Main findings

Perceived barriers and concerns exist concerning the appropriate use of ADs in the confirmatory setting for key stakeholders in clinical trials research. Stakeholder perceptions about barriers are largely consistent across sectors, with a few exceptions that reflect differences in organisational funding structures, experiences, and the nature of study interventions. For example, the lack of bridge funding accessible to UK CTUs in the form of small grants to support design developmental work of time-consuming and complex ADs was reported as the major stumbling block to the routine use of ADs.

The most important cross-sector barriers appear to be connected to the dearth of practical implementation knowledge and experience, lack of applied training, and paucity of implemented case studies of ADs to facilitate practical learning and problem-solving. This is intertwined with the amount of time and effort required in the planning of ADs. Moreover, both the private sector and UK CTUs voiced concerns that they are under immense pressure to deliver on other competing priorities that are based on simpler traditional mainstream trial designs. Hence, they have limited time to support complex ADs, even when they are appropriate.

### Implications for practice

There is cross-sector and multidisciplinary interest in the use of ADs when appropriate to answer research questions. The benefits of ADs can only be reaped when key barriers to their use are adequately addressed.

First, there seems to be a strong need on the part of public funders to address sources of funding accessible to UK CTUs wishing to support the use of relevant, complex, and time-consuming ADs. For instance, the MAMS design requires in-depth statistical simulations and time commitment. This developmental stage is often unfunded, with researchers taking risks betting on uncertain future success of research grants. Hence, given the high risk involved, UK CTUs may be reluctant to support such ADs, even when they are more relevant to answer research questions efficiently. Although in the UK, the NIHR provides infrastructure funding accessible to accredited CTUs [21], this funding is often used for other purposes, such as meeting contractual obligations of staff who may not receive funding between studies. There is an opportunity for the NIHR and MRC to create a small funding stream to support the design of time-consuming designs provided the research questions meet their priority needs, and there is a strong design rationale. The funding should be conditional on open access publication of design-related material such as software programs to enhance planning of related future trials. We would also encourage the use of ADs, which are simple to implement within the existing scope of public funding models for fixed sample size designs, such as sample size reviews and futility assessments.

Most of the important barriers reported here are associated with a lack of practical knowledge and experience among key stakeholders. Numerous theoretical developments have occurred in ADs, and more are needed to address unknowns. However, what is lacking is a translational framework to enhance the use of the ADs in practice. We encourage accessible publication of case studies of successful and unsuccessful ADs with related materials. These publications should encompass aspects such as rationale and design; statistical and practical challenges, and how they are resolved; implementation resources; lessons learned; regulatory, data management and communication hurdles, and how these are resolved; and other facilitators to successful implementation. Learning from researchers or organisations who are routinely implementing ADs is paramount. Most importantly, a need exists for a focal group of practical experts who are publicly funded to support those CTUs with little practical expertise wishing to implement ADs. Furthermore, such experts should provide practical training accessible on ADs to UK CTUs. Although an initiative exists through the MRC Hubs for Trials Methodology Research Network AD Working Group [22], some trialists viewed it as being more theoretically oriented [12]. Most importantly, it seems that the time is upon us for close collaboration in the practical implementation of ADs.

We strongly encourage researchers who receive public funding for AD-related methodological research to produce open access resources such as free-to-use software or code to implement the methods developed. This would facilitate the application of the methods. A recommendation also is that CTUs receiving AD-related bridge or research funding should form a compendium of case studies for publication. Open-access publication of research outputs and resources such as in monographs is important. This could be an important resource aimed at reducing research waste and improving the appropriate conduct of adaptive trials. Such knowledge-sharing would be helpful for applied knowledge transfer.

Concerns regarding the robustness of ADs in decision-making and their credibility to change practice when trials are stopped early are real and should be addressed. Even though there are multi-dimensional aspects to these concerns, transparent and adequate reporting of trial conduct may alleviate some of the concerns. For instance, suboptimal reporting of appropriate statistical methods to obtain unbiased trial results (point estimates, CIs and P-values) following an AD may influence consumers of research findings to view them with suspicion [23–25]. There is a need for a CONSORT statement tailored for ADs to enhance their reporting and conduct. We support recent related initiatives proposing some adaptive trial aspects, which should be reported by researchers [26]. Case studies of ADs investigating a wide range of interventions published in ‘high impact’ journals and their influence on clinical practice could also help to convince sceptical research consumers.

Like any new methods, the use of ADs in confirmatory trials is bound to raise anxiety among some researchers. Some of this anxiety could be alleviated by a cross-disciplinary, consensus guidance document on ADs well-crafted to addressing pertinent issues in confirmatory trials. For instance, the design of complex interventions has gone through a similar phase, but the emergence of related guidance documents improved researchers’ receptiveness towards their conduct [27]. Most importantly, there is a need for a troubleshooting toolkit with pertinent design-specific questions, which trialists need to ask themselves when considering ADs. This could be helpful in the appropriate planning of ADs.

Optimal description of any proposed AD by researchers to key stakeholders (reviewers, funders/sponsors, collaborators and regulators) where appropriate is fundamental; its rationale within the context of the research objectives, potential benefits compared to mainstream traditional approach, scope, decision criteria to guide the adaptation and decision-making process, variable costs and trial durations, measures to minimise operational bias and control of statistical properties (type I error rate, power and inference), among others should be addressed. For instance, our preceding research [12] found that the fear of risking regulatory approval does not necessarily reflect regulatory perspective but is mostly an artefact of inadequate description of the proposed AD and its suitability to address the research question(s). There are encouraging indications that regulatory receptiveness to the appropriate use of ADs is positive, with improving awareness and experiences, particularly with respect to scientific advice and review of AD proposals [5, 12, 28, 29]. In this regard, as reflected in our preceding research [12], we encourage funders to modify their grant application forms to facilitate adequate description of AD-related aspects. This could be achieved by allowing clinical trialists to add specific relevant AD material as appendices.

Periodic ‘refresher training’ of public funding boards and panel members prior to their commissioning meeting may help alleviate a lack of awareness of the acceptable scope of ADs, when they are appropriate, and their benefits in confirmatory trials. We believe the experience of funding boards and panels can only be improved when researchers put forward more appropriate AD-related grant proposals for consideration. A positive change in attitudes and receptiveness towards appropriate use of ADs by public funders is an encouraging opportunity, which should be exploited by researchers.

The challenges faced by researchers in developing widely-acceptable decision-making criteria at the design stage to inform the adaptation process can be alleviated through multidisciplinary engagement and discussions during planning. This process should include close discussions among key stakeholders such as trial statisticians, clinicians, patient representatives, clinical peer advocate groups, and regulators. Our preceding study summarises some additional facilitators to the appropriate use of ADs [12].

### Interpretation of the findings

This study is based on themes generated from cross-sector and cross disciplinary in-depth interviews of key stakeholders involved in clinical trials research. Notably, the surveys explored the perceptions of funders and trialists towards ADs, predominantly in the UK confirmatory setting. Most importantly, this is the first study that employed Rating Scale Modelling to rank barriers in order of perceived importance to address the research question.

Some of our findings on barriers are consistent with the literature [4, 6, 9, 11]. The most top-rated barriers, such as lack of practical knowledge, lack of applied training, time constraints to support planning, research teams preferring traditional mainstream designs to ADs, and limited access to case studies about ADs to facilitate practical training were reported in both the private and public sectors. In contrast, some barriers such as inadequate data management infrastructure to support ADs and associated practical complexities during planning were viewed by private sector organisations as more important than in the public sector. This could be partly explained by differences in experiences in the conduct of ADs – hence, a differential awareness of the practical challenges faced. This could also be due to the differences in the type of interventions under investigation and the regulatory framework.

It should be noted that our findings are based on moderate response rates across sectors. Most recent surveys of UK CTUs observed response rates of 38 % [30] and ranged from 25 % to 67 % [31]. The private sector organisations we had contact with were invited to take part. The number of responders was similar to previous research by the AD Working Group (USA) although they achieved a 100 % questionnaire response rate over a 1-year period [6]. To the best of our knowledge, we are not aware of previous research surveying public funders. Bearing all this in mind and based on email replies from non-responders (UK CTUs and public funders), the non-responders are likely different from the responders. For instance, non-responders seem more likely to be unfamiliar with ADs or do not view ADs as a priority. Hence, some of our findings on barriers, such as awareness of ADs, their benefits and acceptable scope in confirmatory setting, and lack of statistical expertise highlighted during interviews [12], could be underestimated. On the other hand, one could argue that such findings are conservative.

## Conclusions

A general multi-disciplinary and cross-sector interest exists for the appropriate use of ADs. However, there are still persistent and important perceptions of organisational and individual roadblocks hampering the appropriate uptake of ADs in clinical trial practice. The lack of bridge funding accessible to UK CTUs wishing to support developmental design work seems to be an important obstacle requiring redress. A cross-sector collaboration and paradigm shift towards translational applied training, and accessible publication of case studies on ADs are paramount in addressing the dearth of knowledge and experience. Most importantly, a troubleshooting toolkit of key design-specific questions researchers need to ask themselves when considering ADs, transparent and adequate reporting of the conduct of adaptive trials, and a multidisciplinary consensus guidance document on the acceptable scope of ADs in confirmatory trials are all required to facilitate their appropriate use.

## Additional files

Additional file 1:
**Supplementary summary data on UK Clinical Trials Unit (CTU) perceptions of important barriers to adaptive designs (ADs) use in confirmatory trials; Summary statistics.** (PDF 88 kb) Additional file 2:
**Supplementary summary data on public funder perceptions of important barriers to adaptive designs (ADs) use in confirmatory trials; Summary statistics.** (PDF 75 kb) Additional file 3:
**Supplementary summary data on private sector organisations’ perceptions of important barriers to adaptive designs (ADs) use in confirmatory trials; Summary statistics.** (PDF 157 kb) Additional file 4:
**Cross-sector perceptions of concerns towards adaptive designs (ADs) use in confirmatory trials; Summary statistics.** (PDF 33 kb)

## Abbreviations

AD: adaptive design
CI: confidence interval
CR UK: Cancer Research United Kingdom
CTU: Clinical Trials Unit
CRO: Contract Research Organisation
EMA: European Medicines Agency
EME: Efficacy Mechanism and Evaluation
FDA: Food and Drug Administration
GSD: group sequential design
HTA: Health Technology Assessment
NIHR: National Institute for Health Research
MHRA: Medicines and Healthcare products Regulatory Agency
MRC: Medical Research Council
NHS: National Health Service
R & D: Research and Development
RfPB: Research for Patient Benefit
SSR: sample size re-estimation
UK CRC: United Kingdom Clinical Research Collaboration
MAMS: multi-arm multi-stage
