# Supplementary material for: Cross-sector surveys assessing perceptions of key stakeholders towards barriers, concerns and facilitators to the appropriate use of adaptive designs in confirmatory trials
Source: Trials. 2015 Dec 23;16:585. doi: 10.1186/s13063-015-1119-x (PMC4690427; doi:10.1186/s13063-015-1119-x)
Supplement: Additional file 1: — Supplementary summary data on UK Clinical Trials Unit (CTU) perceptions of important barriers to adaptive designs (ADs) use in confirmatory trials; Summary statistics. (PDF 88 kb) [file 13063_2015_1119_MOESM1_ESM.pdf]

Supplementary summary data on UK CTUs' perceptions of important barriers to ADs use in confirmatory trials

Barrier	Perceived importance				Relative importance parameter (95% CI)	Rank
	Not important	Somewhat important	Moderately important	Extremely important		
Lack of bridge funding required to support design work of time consuming and complex ADs	3(12%)	10(40%)	4(16%)	8(32%)	-1.05(-1.59 to -0.52)	1
Lack of practical implementation knowledge	5(20%)	5(20%)	9(36%)	6(24%)	-1.02(-1.56 to -0.49)	2
Lack of practical hands-on experience	5(20%)	5(20%)	9(36%)	6(24%)	-1.02(-1.55 to -0.48)	3
Research team being more comfortable with the conventional mainstream designs compared to ADs	3(12%)	10(40%)	6(24%)	6(24%)	-0.90(-1.43 to -0.37)	4
Difficulties in marketing ADs to key stakeholders in trials research (such as collaborators, funders, and regulators)	5(20%)	7(28%)	8(32%)	5(20%)	-0.72(-1.25 to -0.19)	5
Amount of work and effort required at the design or planning stage	6(24%)	7(28%)	6(24%)	6(24%)	-0.67(-1.20 to -0.14)	6
Lack of time to support planning in relation to other competing conventional mainstream design priorities	5(20%)	8(32%)	7(28%)	5(20%)	-0.67(-1.20 to -0.14)	7
Lack of applied training to facilitate practical implementation	5(20%)	7(28%)	10(40%)	3(12%)	-0.60(-1.13 to -0.07)	8
Insufficient access to case studies to facilitate practical learning	4(16%)	10(40%)	7(28%)	4(16%)	-0.60(-1.12 to -0.07)	9
Practical complexities during trial conduct for successful implementation	5(20%)	9(36%)	8(32%)	3(12%)	-0.47(-1.00 to 0.06)	10
Statistical complexities during planning (such as simulation work)	8(32%)	4(16%)	10(40%)	3(12%)	-0.41(-0.94 to 0.12)	11
Difficulties in setting up acceptable upfront decision making criteria to guide the adaptation	10(40%)	4(16%)	6(24%)	5(20%)	-0.27(-0.80 to 0.26)	12
Lack of capacity of proposal developers with basic knowhow	6(24%)	11(44%)	6(24%)	2(8%)	-0.10(-0.64 to 0.43)	13
Costing complexities on grant application	6(24%)	12(48%)	4(16%)	3(12%)	-0.10(-0.64 to 0.44)	14
Lack of awareness of which scope of ADs are acceptable in confirmatory trials	6(24%)	12(48%)	5(20%)	2(8%)	-0.03(-0.57 to 0.51)	15
Lack of awareness of when ADs are appropriate	8(32%)	10(40%)	6(24%)	1(4%)	0.18(-0.36 to 0.73)	16
Worry about the impact of stopping early on full-time research staff employment contracts	10(40%)	8(32%)	4(16%)	3(12%)	0.21(-0.34 to 0.76)	17
Unfamiliarity with key implementation resources such as validated statistical software	8(32%)	11(44%)	6(24%)	-	0.34(-0.22 to 0.90)	18
Fear of regulatory reluctance and jeopardising chances of obtaining regulatory approval due to the use of an AD	11(44%)	6(24%)	7(28%)	1(4%)	0.37(-0.19 to 0.93)	19
Inadequate data management support infrastructure for timely capturing, cleaning and transfer for decision making as part of the adaptation	12(48%)	6(24%)	4(16%)	3(12%)	0.39(-0.17 to 0.96)	20
Statistical complexities during implementation (such as analysis and reporting)	9(36%)	10(40%)	6(24%)	-	0.42(-0.15 to 0.98)	21
Lack of knowledge to use existing validated statistical software	9(36%)	11(44%)	4(16%)	1(4%)	0.42(-0.14 to 0.99)	22
Lack of statistical expertise	9(36%)	10(40%)	6(24%)	-	0.43(-0.13 to 0.99)	23
Lack of awareness of benefits of ADs	12(48%)	6(24%)	7(28%)	-	0.60(0.02 to 1.18)	24

Tension during early stopping decision making among key decision makers (such as IDMC and funders)	15(60%)	7(28%)	3(12%)	-	1.32(0.66 to 1.98)	25
Previous negative experiences during implementation	19(76%)	3(12%)	2(8%)	1(4%)	1.79(1.04 to 2.53)	26
Previous negative experiences with ADs based on funders/reviewers comments	19(76%)	5(20%)	1(4%)	-	2.15(1.32 to 2.99)	27
