# Supplementary material for: Cross-sector surveys assessing perceptions of key stakeholders towards barriers, concerns and facilitators to the appropriate use of adaptive designs in confirmatory trials
Source: Trials. 2015 Dec 23;16:585. doi: 10.1186/s13063-015-1119-x (PMC4690427; doi:10.1186/s13063-015-1119-x)
Supplement: Additional file 2: — Supplementary summary data on public funder perceptions of important barriers to adaptive designs (ADs) use in confirmatory trials; Summary statistics. (PDF 75 kb) [file 13063_2015_1119_MOESM2_ESM.pdf]

Supplementary summary data on Public Funders' perceptions of important barriers to ADs use in confirmatory trials

Barrier	Perceive importance				Relative importance parameter (95% CI)	Rank
	Not important	Somewhat important	Moderately important	Extremely important		
Funding panel board members being more comfortable with traditional mainstream designs compared to ADs	7(11%)	17(27%)	21(33%)	19(30%)	-0.58 (-0.93 to -0.24)	1
Funding board generally being risk averse to fund complex ADs associated with high financial uncertainty	10(17%)	16(27%)	15(25%)	19(32%)	-0.45 (-0.81 to -0.10)	2
Decision making criteria to guide the adaptation not well described	7(11%)	18(29%)	26(41%)	12(19%)	-0.33 (-0.67 to 0.02)	3
Rationale for ADs not well explained in the grant application	5(8%)	25(40%)	18(29%)	15(24%)	-0.32 (-0.66 to 0.02)	4
Lack of expertise of reviewers of ADs to help funding panel boards during grant review process	8(14%)	19(32%)	21(36%)	11(19%)	-0.22 (-0.57 to 0.14)	5
Lack of commissioning experience of ADs	8(14%)	17(30%)	23(40%)	9(16%)	-0.19 (-0.55 to 0.17)	6
The type AD proposed and its scope not well described in the grant application	8(13%)	20(32%)	23(37%)	11(18%)	-0.14 (-0.48 to 0.21)	7
Lack of awareness of which scope of ADs are acceptable in confirmatory trials	8(13%)	21(34%)	25(40%)	8(13%)	-0.03 (-0.38 to 0.31)	8
Lack of awareness of when ADs are appropriate	11(18%)	21(34%)	18(29%)	12(19%)	0.02 (-0.32 to 0.37)	9
Inadequate description of the costing scenarios of ADs in the grant application	7(11%)	25(41%)	22(36%)	7(11%)	0.04 (-0.31 to 0.39)	10
Lack of awareness of benefits of ADs	16(25%)	17(27%)	17(27%)	14(22%)	0.18 (-0.16 to 0.52)	11
Difficulties in drawing up flexible contractual agreements suitable for ADs	16(28%)	14(24%)	22(38%)	6(10%)	0.41 (0.05 to 0.77)	12
Tension during early stopping decision making of ADs among key decision makers	12(21%)	26(46%)	14(25%)	4(7%)	0.63 (0.25 to 1.00)	13
Negative attitudes towards ADs among some funding panel board members	22(37%)	22(37%)	8(13%)	8(13%)	0.97 (0.60 to 1.34)	14

Note: The number of participants in the denominator varies due to the exclusion of respondents who were not able to answer certain items
