# Supplementary material for: Cross-sector surveys assessing perceptions of key stakeholders towards barriers, concerns and facilitators to the appropriate use of adaptive designs in confirmatory trials
Source: Trials. 2015 Dec 23;16:585. doi: 10.1186/s13063-015-1119-x (PMC4690427; doi:10.1186/s13063-015-1119-x)
Supplement: Additional file 3: — Supplementary summary data on private sector organisations’ perceptions of important barriers to adaptive designs (ADs) use in confirmatory trials; Summary statistics. (PDF 157 kb) [file 13063_2015_1119_MOESM3_ESM.pdf]

## Supplementary summary data on private sector's perceptions of important barriers to ADs use in confirmatory trials

| Barrier | Perceived importance | | | | Relative importance parameter (95% CI) | Rank |
|---|---|---|---|---|---|---|
| | Not important | Somewhat important | Moderately important | Extremely important | | |
| Lack of practical implementation knowledge | 1(8%) | 3(23%) | 5(38%) | 4(31%) | -1.44(-2.42 to -0.46) | 1 |
| Lack of time to support planning in relation to other competing conventional mainstream design priorities | 1(8%) | 6(46%) | 2(15%) | 4(31%) | -1.24(-2.20 to -0.27) | 2 |
| Practical complexities during trial conduct for successful implementation | 1(8%) | 3(23%) | 6(46%) | 3(23%) | -1.19(-2.16 to -0.23) | 3 |
| Inadequate data management support infrastructure for timely capturing, cleaning and transfer for decision making as part of the adaptation | 3(23%) | 5(38%) | 2(15%) | 3(23%) | -1.07(-2.03 to -0.12) | 4 |
| Lack of applied training to facilitate practical implementation | 2(15%) | 2(15%) | 7(54%) | 2(15%) | -0.98(-1.93 to -0.03) | 5 |
| Lack of practical experiences | 1(8%) | 4(31%) | 6(46%) | 2(15%) | -0.92(-1.87 to 0.02) | 6 |
| Insufficient access to case studies to facilitate practical learning | 2(15%) | 5(38%) | 4(31%) | 2(15%) | -0.78(-1.72 to 0.16) | 7 |
| Research team being more comfortable with the conventional mainstream designs compared to ADs | 3(23%) | 2(15%) | 5(38%) | 3(23%) | -0.74(-1.68 to 0.20) | 8 |
| Lack of awareness of which scope of ADs are acceptable in confirmatory trials | 1(8%) | 4(31%) | 6(46%) | 2(15%) | -0.64(-1.57 to 0.30) | 9 |
| Amount of work and effort required at the design or planning stage | 4(31%) | 4(31%) | 2(15%) | 3(23%) | -0.54(-1.47 to 0.40) | 10 |
| Fear of regulatory reluctance and jeopardising chances of obtaining regulatory approval due to the use of an AD † | 5(38%) | 1(8%) | 3(23%) | 3(23%) | -0.53(-1.46 to 0.40) | 11 |
| Difficulties in marketing ADs to key stakeholders in trials research (such as collaborators, R& D and regulators) | 2(15%) | 6(46%) | 4(31%) | 1(8%) | -0.51(-1.45 to 0.42) | 13 |
| Lack of awareness of when ADs are appropriate | 2(15%) | 5(38%) | 4(31%) | 2(15%) | -0.46(-1.39 to 0.47) | 13 |
| Difficulties in setting up acceptable upfront decision making criteria to guide the adaptation | 2(15%) | 5(38%) | 2(15%) | 4(31%) | -0.31(-1.24 to 0.62) | 14 |
| Statistical complexities during planning (such as simulation work) | 3(23%) | 6(46%) | 2(15%) | 2(15%) | -0.09(-1.03 to 0.85) | 15 |
| Lack of bridge funding required to support design work of time consuming and complex ADs † | 6(46%) | 3(23%) | 2(15%) | 1(8%) | -0.04(-0.98 to 0.90) | 16 |
| Difficulties outsourcing expertise to support ADs † | 5(38%) | 3(23%) | 4(31%) | - | 0.20(-0.76 to 1.16) | 17 |
| Tension during early stopping decision making among key decision makers (such as data monitoring committees and sponsors/funders) | 4(31%) | 6(46%) | 1(8%) | 2(15%) | 0.34(-0.64 to 1.31) | 18 |
| Statistical complexities during implementation (such as analysis and reporting) | 5(38%) | 6(46%) | - | 2(15%) | 0.38(-0.60 to 1.36) | 19 |
| Lack of awareness of benefits of ADs | 7(54%) | 2(15%) | 3(23%) | 1(8%) | 0.38(-0.60 to 1.370 | 20 |
| Lack of knowledge to use existing validated statistical software | 6(46%) | 4(31%) | 2(15%) | 1(8%) | 0.50(-0.50 to 1.50) | 21 |

| | | | | | | |
|---|---|---|---|---|---|---|
| Unfamiliarity with key implementation resources such as validated statistical software † | 6(46%) | 3(23%) | 3(23%) | 1(8%) | 0.50(-0.50 to 1.50) | 22 |
| Lack of motivational support from R&D to build an infrastructure to support ADs | 6(46%) | 5(38%) | 2(15%) | - | 0.65(-0.37 to 1.67) | 23 |
| Previous negative regulatory experiences with ADs such as based on regulatory comments or unsuccessful implementation † | 5(38%) | 3(23%) | 2(15%) | - | 0.70(-0.33 to 1.73) | 24 |
| Lack of general expertise around ADs at the trial planning stage | 5(38%) | 4(31%) | 4(31%) | - | 0.71(-0.32 to 1.74) | 25 |
| Complexity in deriving the cost of the proposed trial † | 6(46%) | 3(23%) | 2(15%) | 1(8%) | 0.91(-0.16 to 1.99) | 26 |
| Previous negative experiences with ADs during implementation † | 7(54%) | 2(15%) | 2(15%) | - | 0.97(-0.12 to 2.05) | 27 |
| Insufficient financial support from R&D to build an infrastructure to support ADs | 8(62%) | 2(15%) | 2(15%) | 1(8%) | 1.00(-0.09 to 2.10) | 28 |
| Lack of statistical expertise | 8(62%) | 2(15%) | 3(23%) | - | 1.09(-0.03 to 2.20) | 29 |
| Worry about the impact of stopping early on staff contracts † | 10(77%) | 2(15%) | - | - | 3.16(1.01 to 5.31) | 30 |

† Some respondents selected not applicable to their organisation
