# Supplementary material for: Cross-sector surveys assessing perceptions of key stakeholders towards barriers, concerns and facilitators to the appropriate use of adaptive designs in confirmatory trials
Source: Trials. 2015 Dec 23;16:585. doi: 10.1186/s13063-015-1119-x (PMC4690427; doi:10.1186/s13063-015-1119-x)
Supplement: Additional file 4: — Cross-sector perceptions of concerns towards adaptive designs (ADs) use in confirmatory trials; Summary statistics. (PDF 33 kb) [file 13063_2015_1119_MOESM4_ESM.pdf]

Cross-sector perceptions of concerns towards ADs use in confirmatory trials

Concern	Perceived level of concern					Relative concern parameter (95% CI)	Rank
	Not at all	Slightly	Somewhat	Moderately	Extremely		
**UK CTUs**							
Efficacy early stopping of trials	7(28%)	4(16%)	4(16%)	4(16%)	6(24%)	-0.35(-0.76 to 0.07)	1
Robustness of AD methodology to influence policy decision making when trials are stopped early	2(8%)	8(32%)	8(32%)	5(20%)	2(8%)	-0.26(-0.67 to 0.15)	2
Non-inferiority early stopping of trials	9(36%)	2(8%)	5(20%)	4(16%)	5(20%)	-0.15(-0.56 to 0.26)	3
Fear of introducing operational bias	3(12%)	11(44%)	4(16%)	4(16%)	3(12%)	-0.10(-0.51 to 0.31)	4
Impact of ADs on secondary trial objectives when trials are stopped early	2(8%)	12(48%)	6(24%)	3(12%)	2(8%)	-0.01(-0.43 to 0.40)	5
Acceptability of the findings from ADs by the research community or regulators to change practice	5(20%)	9(36%)	6(24%)	3(12%)	2(8%)	0.13(-0.29 to 0.55)	6
Potential change in the population during the course of an adaptive trial and its impact on interpretation of the findings	7(28%)	9(36%)	2(8%)	5(20%)	2(8%)	0.23(-0.20 to 0.66)	7
Futility stopping of trials for futility	10(40%)	4(16%)	8(32%)	2(8%)	1(4%)	0.51(0.07 to 0.95)	8
**Private sector**							
Early stopping of trials for non-inferiority	2(15%)	3(23%)	3(23%)	4(31%)	1(8%)	-0.39(-0.99 to 0.20)	1
Impact of ADs on secondary trial objectives when trials are stopped early	2(15%)	5(38%)	1(8%)	4(31%)	1(8%)	-0.24(-0.84 to 0.37)	2
Fear of introducing operational bias	1(8%)	8(62%)	-	2(15%)	2(15%)	-0.22(-0.82 to 0.39)	3
Early stopping of trials for efficacy	3(23%)	3(23%)	2(15%)	5(38%)	-	-0.16(-0.76 to 0.45)	4
Potential change in the population during the course of an adaptive trial and its impact on interpretation of the findings	3(23%)	4(31%)	2(15%)	3(23%)	1(8%)	-0.09(-0.70 to 0.52)	5
Robustness of AD methodology to influence policy decision making when trials are stopped early	3(23%)	4(31%)	2(15%)	4(31%)	-	-0.00(-0.62 to 0.61)	6
Acceptability of the findings from ADs by the research community or regulators in order to change practice	3(23%)	5(38%)	2(15%)	3(23%)	-	0.22(-0.40 to 0.85)	7
Early stopping of trials for futility	7(54%)	1(8%)	3(23%)	2(15%)	-	0.87(0.18 to 1.55)	8
**Public Funders**							
Robustness of AD methodology to influence policy decision making when trials are stopped early	7(10%)	8(12%)	23(34%)	15(22%)	9(13%)	-0.46(-0.73 to -0.19)	1
Acceptability of the findings from ADs by the research community or regulators in order to change practice	5(7%)	12(18%)	18(26%)	19(28%)	7(10%)	-0.43(-0.70 to -0.16)	2
Impact of ADs on secondary trial objectives when trials are stopped early	7(10%)	17(25%)	20(29%)	14(21%)	4(6%)	-0.09(-0.35 to 0.18)	3
Non-inferiority early stopping of trials	13(19%)	15(22%)	13(19%)	11(16%)	9(13%)	-0.07(-0.34 to 0.20)	4
Fear of introducing operational bias	10(15%)	14(21%)	22(32%)	11(16%)	4(6%)	0.04(-0.23 to 0.31)	5

Potential change in the population during the course of an adaptive trial and its impact on interpretation of the findings	10(15%)	21(31%)	17(25%)	9(13%)	4(6%)	0.17(-0.11 to 0.44)	6
Efficacy early stopping of trials	13(21%)	20(33%)	14(23%)	10(16%)	4(7%)	0.25(-0.02 to 0.52)	7
Futility early stopping of trials	22(32%)	17(25%)	10(15%)	7(10%)	5(7%)	0.58(0.29 to 0.86)	8
